# Getting the most out of dual-chamber leadless pacing: how to prolong battery longevity?

**DOI:** 10.1093/europace/euaf073

**Published:** 2025-07-02

**Authors:** Haran Burri

**Affiliations:** Cardiac Pacing Unit, Department of Cardiology, University Hospital of Geneva, rue Gabrielle Perret Gentil 4, Geneva 1211, Switzerland

## Abstract

**Graphical Abstract:**
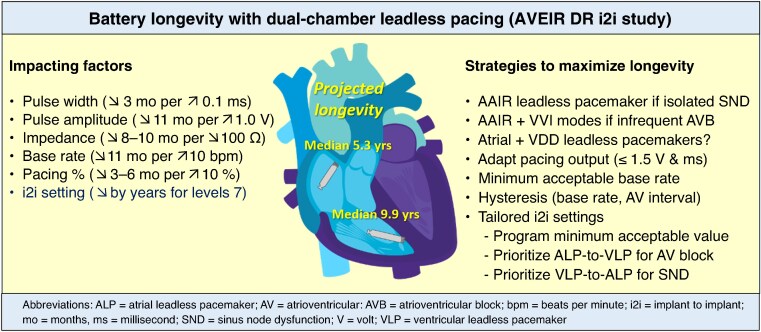

**This editorial refers to ‘Battery longevity of a helix-fixation dual-chamber leadless pacemaker results from the AVEIR DR i2i study’ by V.Y. Reddy *et al.*, https://doi.org/10.1093/europace/euaf074**

The atrial leadless pacemaker (ALP) is a milestone in pacing therapy as it has made leadless atrial pacing possible. Furthermore, > 96% atrioventricular synchrony can be achieved by implant-to-implant (i2i) transmission with a ventricular leadless pacemaker (VLP).^1,2^

Battery longevity of leadless pacemakers is a key consideration, due to increasing life expectancy, indications in younger patients, and complexity/costs related to generator change. Furthermore, the longevity of the current dual-chamber leadless system is determined by that of its shortest-lived component. The ALP has 72% of the battery capacity of the VLP (174 vs. 241 mAh) as a consequence of its shorter length. As a comparison, the battery of the Medtronic (Minneapolis, USA) Micra AV2 has a capacity of 142 mAh (with a projected median longevity estimate of 10.9 years when programmed to VDD mode^3^). Estimates of battery longevity based on simulations (i.e. not from actual clinical data) are available (see *Table 1*).^1^ It is thereby apparent that programming dual-chamber pacing with i2i communication (as opposed to single-chamber pacing) has a considerable impact on device longevity.

**Table 1:** Projected service life estimates of AVEIR leadless pacemakers (nominal longevity—beginning of service to recommended replacement time)

Atrial device			Longevity (Year) Impedence			
Base rate	Amplitude/0.4 ms	%Pacing	400 Ω		500 Ω	
			AAIR	DDDR	AAIR	DDDR
50 bpm	1.25 V	100%	11.9	7.1	12.8	7.9
		50%	14.5	8.1	15.2	8.8
		0%	18.7	9.3	18.9	10
50 bpm	2.5 V	100%	6.3	4.7	7.2	5.3
		50%	9.5	6.2	10.5	6.9
		0%	18.7	9.3	18.9	10
60 bpm	1.25 V	100%	10.4	6.1	11.2	6.8
	2.5 V	100%	5.4	3.9	6.1	4.3
	5.0 V	100%	1.5	1.4	1.8	1.6

Ventricular device			Longevity (Years) Impedence			
Base rate	Amplitude/0.4 ms	%Pacing	500 Ω		600 Ω	
			VVIR	DDDR	VVIR	DDDR
50 bpm	1.25 V	100%	18.4	11.4	19.4	12.2
		50%	21.8	12.7	22.6	13.5
		0%	26.9	14.3	27.1	15.0
50 bpm	2.5 V	100%	10.4	7.7	11.5	8.5
		50%	15.0	10.0	16.1	10.9
		0%	26.9	14.3	27.1	15.0
60 bpm	1.25 V	100%	16.1	9.8	17.1	10.5
	2.5 V	100%	8.9	6.6	9.9	7.3
	5.0 V	100%	2.7	2.5	3.2	2.8

Adapted from supplementary material in Knops *et al*.^1^

In this edition of the journal, Reddy *et al*.^4^ provide an in-depth evaluation of the projected battery longevities of the AVEIR DR system (Abbott, Sylmar, USA). In 302 patients with *de novo* dual-chamber systems successfully implanted in the Aveir DR i2i Study, estimated remaining battery longevity was calculated by the programmer using the battery consumption between 6 and 12 months (data were cleared at 6 months after initial optimization), programmed settings, pacing impedance, pacing percentage, and sensed/paced event rate. The median total ALP and VLP longevities were 5.3 and 9.9 years, respectively and were impacted by the factors shown in the graphical abstract. The greatest impact was for the i2i setting, which was non-linear with a reduction by approximately 2 years for ALPs and 3.5 years for VLPs when programming i2i transmission setting from 6 to 7.

Even though these results are only projected estimates and the actual device longevities will still have to be proven at follow-up, they provide us with valuable data to devise strategies to prolong battery life.

## The cost of implant-to-implant communication

The AVEIR DR system uses bidirectional conductive i2i communication through blood and myocardial tissue by a short series of subthreshold electrical pulses which are sent immediately before delivery of pacing and immediately after a sensed event to allow the receiving device to initiate refractory periods and timers. Transmission uses the same pacing and sensing circuits as for antibradycardia pacing. Inter-device distance is a key factor (maximum recommended button-to-button distance of 70 mm), and to a lesser extent angulation between devices (best if aligned). Transmission settings (from 1 to 7, nominal 4) are programmed separately for the ALP and the VLP, either automatically or manually. Changing the i2i setting impacts pulse output as well as the sensitivity level of the receiving device (independently from the antibradycardia function). Increasing transmission settings between 1 and 6 results in an increase in pulse width with a decrease in longevity in the order of months; for setting 7, pulse amplitude is increased, shortening battery longevity by years. In the AVEIR i2i study, the ALP-to-VLP and VLP-to-ALP i2i settings programmed at implant were 5.2 ± 1.2 and 5.6 ± 1.2, and at 6 months they were slightly reduced to 4.5 ± 1.4 and 5.1 ± 1.4, respectively. Interestingly, ∼20% patients had <70% successful i2i communication at discharge, which decreased to only ∼5% at 3 months, with a *decrease* in the programmed i2i setting compared to implant in 50–60% of patients.^5^ This may be due to changes in posture (with dominant recumbency before discharge, whereas variable postures were adopted during daily activities). It is also possible that a reduction in pacing impedance (which is observed over follow-up^1^) results in better i2i communication, albeit at the expense of greater current drain.

It is important to note that battery life is impacted by the number of sensed events, which trigger i2i transmissions. Thus, intrinsic heart rate and mode switch episodes (where atrial non-refractory sensed events are transmitted by the ALP) are contributing factors which impact device longevity.

## Currently available strategies to maximize longevity

The most efficient strategy is to avoid i2i communication altogether:

1. Implantation of standalone ALPs for isolated sinus node dysfunction (SND) is being increasingly adopted. Following the DANPACE trial, AAIR pacing came into disfavour due to the more frequent requirement for reintervention and the slightly higher incidence of atrial fibrillation compared to DDDR pacing, even though there were no differences in hard endpoints (mortality, heart failure hospitalization, or stroke).^6,7^ However, adding a VLP is less problematic than adding a ventricular lead for a standard pacemaker due to possible venous access issues, also bearing in mind the risk of infection related to opening the pocket. If patients are well selected for AAIR pacing, the risk of atrioventricular block and of syncope is low. In the DANPACE trial, patients had to have a QRS <0.12 s and 1:1 atrioventricular conduction during pacing at 100 bpm at implantation to qualify for the AAIR arm.^6^ After a follow-up of 5.5 ± 2.6 years, only 2.7% of patients were upgraded from AAIR to DDDR pacing due to third-degree atrioventricular block, and 3.9% of patients who were upgraded had syncope of any cause.^8^ Nevertheless, in selected cases, an implantable loop recorder may be an option to monitor atrioventricular conduction.
2. The Micra AV (Medtronic, MN, USA) provides VDD pacing by detecting atrial mechanical contraction.^9^ It may potentially be used in combination with an ALP to provide atrioventricular pacing without i2i communication. However, atrioventricular synchrony may be lower than using a DDD system, and it is unknown if atrial pacing (rather than sensing) impacts the ability of the Micra AV2 to detect atrial contraction.
3. In case of infrequent atrioventricular block, the ALP may be programmed to AAI(R) and the VLP to backup VVI at a lower baseline rate. The AAI(R)+VVI mode does this automatically (at a nominal rate of -5 bpm) and activates a novel crosstalk protection feature that does not rely on i2i communication. The algorithm is still pending approval in Europe but will be able to be implemented later to systems which are already implanted via a software update on the Abbott Merlin programmer.

Tailored programming for each patient is paramount to save battery life. The pulse amplitude should ideally be programmed to ≤1.5 V, as this allows the leadless pacemaker to use a more efficient power regulator mode which translates to notable longevity benefits (personal communication). In patients with isolated SND, ALP-to-VLP i2i communication is relatively superfluous because of intrinsic conduction, and the programmed setting may be minimized to save battery. However, VLP-to-ALP communication should be prioritized in SND patients as the ALP will not deliver pacing unless VLP-to-ALP transmission occurs (to mitigate the risk of asynchronous pacing between the chambers). Conversely, in patients with atrioventricular block without SND, ALP-to-VLP communication should be prioritized.

Programming rate response increases battery drain due to increase in heart rate and pacing percentage, rather than battery consumption of the temperature sensor which is insignificant (personal communication).

Programming should be individualized for each patient regarding pacing mode, i2i settings, base rate, hysteresis (rate and atrioventricular delay), and pacing output.^10^

## Future perspectives

The AVEIR DR system is still in its first generation and is very likely to evolve with enhanced device longevity due to new battery technology, methods to increase pacing impedance, and novel algorithms (e.g. pacing-avoidance modes or automatic threshold measurements). Harvesting of kinetic energy has been contemplated, but this requires that the mechanical components last for at least as long as the rechargeable battery, and for longer than current batteries.

A single-unit DDD leadless pacemaker is in development by Medtronic, which will no longer require i2i transmission. However, dual-chamber pacing and sensing from a single device will bear a cost on battery longevity, and it remains to be seen how this will compare to dual-device systems with separate batteries.

Finally, there is little doubt that right ventricular pacing (including that from leadless pacemakers) can result in detrimental clinical outcome, to which more physiological alternatives exist such as biventricular pacing and conduction system pacing.^11–13^ Leadless conduction system pacing is being actively investigated and has the potential to offer the best of both worlds, but there are many challenges that need to be overcome before this modality becomes reality in routine practice.
